# Impact of COVID-19 Pandemic on Academic Activity and Health Status among Romanian Medical Dentistry Students; A Cross-Sectional Study

**DOI:** 10.3390/ijerph18116041

**Published:** 2021-06-04

**Authors:** Raluca Iurcov, Lavinia-Maria Pop, Magdalena Iorga

**Affiliations:** 1 Dentistry Department, Faculty of Medicine, University of Oradea, 410073 Oradea, Romania; riurcov@uoradea.ro; 2 Faculty of Psychology and Education Sciences, “Alexandru Ioan Cuza” University, 700111 Iasi, Romania; lavinia.pop@student.uaic.ro; 3 Behavioral Sciences Department, “Grigore T. Popa” University of Medicine and Pharmacy, 700115 Iasi, Romania

**Keywords:** dentistry, student, university, satisfaction, evaluation, COVID-19 pandemic, social distance, teachers, fear of infection, online activity

## Abstract

During the first year of the COVID-19 pandemic, dental faculties had to rethink their way of teaching and interacting with students and of delivering solid theoretical knowledge and practical skills to students. Background: The purpose of the study was to assess dentistry students’ opinions about the online activity, together with a self-evaluation of their mental and physical health, during the first wave of the pandemic. Methods: A cross-sectional study was conducted using an online survey. Three hundred and three students, enrolled across all six years of study, were included in the research. Socio-demographic and academic data were collected, along with a self-evaluation of physical and mental status. Some items investigated students’ opinions about distance learning and the impact of that online activity on their achievement. The answers were rated using a five-item Likert-like scale. Data were analyzed using SPSS (v.24). Results: statistical analyses showed that more than 20% of the students strongly agreed with the statement that they felt more anxious and depressed during the first months of the pandemic, and more than 30% were totally satisfied with their relationships with their family members. One-fifth of the respondents declared that they were totally dissatisfied with the relationships with their colleagues and friends. Overall, 50.60% of the students attended the courses/labs in their entirety when they were connected online. Two-thirds of the respondents considered that their practical training was affected due to the online activity, and that not all of the subjects could be taught online. More than half of the respondents agreed that the most objective evaluation method is that of the multiple-choice exams administered at school, and considered that exclusively utilizing online assessments of students encourages unethical behaviors. Age, involvement in online activity, and active participation using video cameras were strongly correlated with satisfaction with academic results. Conclusions: The results of the present study showed that online activity was a good alternative for dentistry students during the pandemic restrictions. The positive aspects, together with the negative consequences, of distance learning should also be taken into consideration by university teachers and academic institutions to improve teaching experiences and to ensure a solid professional formation for dentistry students.

## 1. Introduction

After the outbreak of the pandemic, universities were forced to align with the restrictions imposed by the governments of each country. The rate of spread of the virus in each country has led to various measures, from the total closure of courses to partial closures, depending on the epidemiological data identified at the regional level.

In the academic environment, the preventive measures taken were, in general, to completely stop all activity in the classroom, to move the didactic activity to an online environment, and to avoid coexistence on university campuses.

According to the American Dental Education Association (ADEA), on 15 May 2020, some key changes were implemented in United States (USA) dental training institutions specifically due to the COVID-19 crisis. Students were sent home for a period, and institutions planned to evaluate their return strategies [1]. On 4 December 2020, the American Academy of General Dentistry (AGD) assembled a list of AGD-sponsored and other online education programs available to members to help them through the pandemic period [2]. On 14 October 2020, the ADEA updated the recommendations for higher education programs in dentistry that intended to initiate the return of dentistry students to campus. The new guidelines were proposed in order to avoid risk and the need for crisis management in higher education, to diminish financial losses, to ensure good learning environments, and to support students’ mental health and the well-being of dentistry faculty members [3].

Many studies conducted in the USA one year after the outbreak of the COVID-19 pandemic also pointed out several positive changes among dental faculties, including curricular changes; students’ involvement in frontline healthcare services; and the adoption of new strategies and methods of learning, teaching, and evaluating students. In order to continue the educational process, policy makers, institutions, teachers, and students worked together to cope with the pandemic crisis. 

A swift implementation of creative methods was adopted to avoid disruptions in practical training relating to patients and clinical competencies [4]. In many educational fields, especially among medical specialties, technology-based learning (TB learning) was used as an efficient educational tool. The virtual educational system was found to help dental students improve their clinical skills [5].

Dental treatments were considered to be of very high risk due to aerosol generation, so the greatest challenge for the teaching staff was to decrease the risk of COVID-19 infection while ensuring the continuity and quality of the dental education system. In light of national COVID-19 infection-control recommendations, dental faculties had to suspend in-person instruction, and teaching activity was conducted online. Some researchers who focused on online dental education pointed out the rapid change adopted by dental faculties in many countries, such as the USA [6,7,8,9], Germany [10], Italy [11], China [12,13], Chile [14], Brazil [8,15,16], Pakistan [17], Nepal [18,19], Indonesia [20], India [21,22], Jordan [23], Romania [24,25,26], Portugal [27,28], Cyprus [29], Australia [8,30], and Spain [31]. During the first year of the pandemic, the academic research teams additionally wanted to identify the differences between countries in what aspects of dental education were disrupted. For example, a multi-country survey conducted by Ammar et al. [32] questioned academic teachers working in dental academic institutions across 28 countries about multiple challenges they experienced during the COVID-19 outbreak. The findings showed the need for human, financial, and technical resources to ensure high quality in online schooling, sharing teaching resources and best practices, and training academics.

The nature of teacher–student interactions changed, and the new type of virtual communication had to use more rich visual content, interactive video tools, graphs, or web-based interaction to meet students’ need for interaction but also to help students create their own learning styles. Individual tasks or online working group sessions were implemented. The need for developing practical skills proved that online learning was an efficient tool in the medical sciences, including dental education, during these special pandemic times.

Distance learning has facilitated the ability for students to continue their studies in dentistry, thus creating the opportunity to access information and simulate practical skills. The online development of the didactic activities also required students’ access to audio—video connection means and to a stable connection to the internet. Various studies [33] showed that in addition to the lack of direct teacher–student and student–patient interaction, the online activity should be maintained due to the highlighted benefits, such as increased satisfaction with learning; individual working styles; synchronous or asynchronous support activities, which give students more freedom to organize their learning and free time; and the use of more interactive methods that provoke students to become more involved in activities. In medical specialties, various studies conducted among dentistry students showed less satisfaction with the teacher–student relationship and a strong disagreement with the effectiveness of online classes [16].

In the field of dentistry education, the evaluation of TB teaching and learning in non-prepared situations, such as those imposed by the COVID-19 pandemic, has not been fully investigated in Romania.

In Romania, between March and October 2020, the faculties of dentistry decided to suspend face-to-face courses. Regarding the next academic year, various methods of carrying out teaching activities were proposed (online or on-site), and practical stages with patients were considered a high-risk activity. This study aimed to evaluate dentistry students’ opinions about academic activity after the first wave of the pandemic and to identify different aspects related to online activity among undergraduate dental students.

## 2. Materials and Methods

### 2.1. Participants and Data Collection

The present study was conducted for two weeks, between 18 November and 2 December 2020. The study period represents the middle of the first academic semester that usually starts in Romania on 1 October and lasts until 10 February. During the period that this study was developed, due to the epidemiological conditions and restrictions imposed among all universities in Romania and Europe, the main parts of the academic activities in the medical sciences were conducted online. The dentistry faculty organized only online activity during this first semester, so students were located at home (i.e., foreign students were in their countries of origin and national students were in their native regions).

The questionnaire was created and distributed using the Google Forms application (Alphabet, Mountain View, CA, USA) to all students of the dentistry faculty across all academic years. The participants were informed about the purpose of the study. Filling in the questionnaire was assumed as informed consent to participate in the study. No incentives were offered to students, and they had the ability to withdraw from the research with no consequences. The inclusion criteria were that participants had to be students enrolled in dentistry studies (in years 1 to 6 of study), and the questionnaire had to be submitted before the deadline. Exclusion criteria were students being enrolled in master’s degree or doctoral research programs and/or questionnaires being submitted after the deadline.

**Figure 1:**
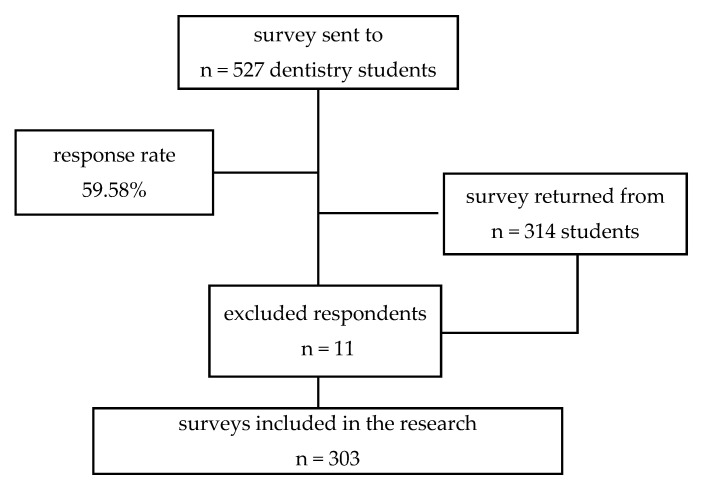
Study profile.

The survey form was sent to 527 students who were registered across all years of study. A total of 314 participants answered the questionnaire before the deadline; after eliminating 11 respondents who declared that they were postgraduate or Ph.D. students, a total of 303 participants were finally included in the research (See Figure 1).

### 2.2. Instruments

After researching the literature about e-learning and online activity during the COVID-19 pandemic, a survey was constructed in order to assess students’ opinions about the educational process during restrictions. The questionnaire contained four parts:

- The first part collected socio-demographic-, academic-, and health status-related data (i.e., age; gender; year of study; being suspected/confirmed as infected with the SARS-Cov2 virus; chronic disease; consumption of cigarettes, alcohol, energy drinks, and drugs; living environment or sharing room during the academic year).
- The second part collected information about students’ physical and mental health status and their fear of infection with COVID-19. Self-rated items were constructed, and answers were rated using a five-item Likert-like scale.
- The third part of the survey inquired about students’ satisfaction with their academic results and relationships with friends, colleagues, and family members, as related to the restrictions imposed by the COVID-19 pandemic.
- The fourth part collected data regarding students’ opinions about their online activities; their practices and habits during lectures; practical stages that were developed online; the teaching and evaluation process; and the impact of online classes on their academic achievement and results, together with ethical concerns about evaluation.
- Self-rated items were constructed, and answers were rated using a five-item Likert-like scale.

### 2.3. Statistical Analysis

All analyses for this research were performed using the IBM Statistical Package for Social Sciences (SPSS) Statistics for Windows, version 24 (SPSS Inc., Chicago, IL, USA). The descriptive statistics of the socio-demographic- and academic-related data were expressed as means (M) and standard deviations (SD), frequencies, and percentages (%). In order to assess comparative results considering gender, marital status, and academic activity, a Mann–Whitney test was performed. The relationship between variables was assessed using Spearman correlations. The level of statistical significance was set at *p* < 0.05.

### 2.4. Ethical Approval

The present study was conducted in accordance with the Declaration of Helsinki, and the protocol was approved by the Ethical Committee of Faculty of Medicine at the University of Oradea, Romania, with the registration No. 12/20.11.2020.

## 3. Results

### 3.1. Socio-Demographic and Academic Data

Socio-demographic-, academic-, and health-related data were collected from all of the participants enrolled in the undergraduate dental program within the University of Oradea at the time of the research. The majority of the respondents were females, which is in congruency with the worldwide gender distribution in medical studies. For descriptive analyses, subjects were presented by year of study; however, this variable was restructured for comparative results as preclinical (years 1, 2, and 3 of study) and clinical (years 4, 5, and 6 of study) academic years. The descriptive analysis of data is presented in Table 1.

### 3.2. Mental Health Status

Social distancing requirements, restrictions against visiting family members and friends, and the interruption of academic activity at the university or in dental clinics were all reported to have had a great impact on the psychological health status of students. Students were also asked about the activities that they engaged in during the first months of the pandemic in order to cope with stress related to the various restrictions and to keep them healthy. More than one-third of the respondents declared that they had watched documentaries, films, and show series (n = 102, 33.66%); more than one-fifth indicated that they had read books or journals (n = 66, 21.78%); a minority of the group declared that they had cooked (n = 37, 12.21%); and some of the respondents said that they had spent their time doing physical activities (n = 36, 11.88%).

Students were asked to self-rate their mental health status regarding the following different problems: sleep, eating, depression, anxiety, and getting bored. The respondents had to indicate their level of agreement with the statements using a five-item Likert-type scale. Answer options included 1—strongly disagree, 2—disagree, 3—neither agree nor disagree, 4—agree, and 5—strongly agree. The students’ scores are detailed in Table 2.

### 3.3. Satisfaction with Health-Status and Relationships with Family, Friends, and Classmates

Six items investigated students’ satisfaction regarding their physical and mental health; their relationships with friends, family members, and colleagues; and their academic results during the first months of COVID-19 restrictions. The answers were rated using a five-item Likert-type scale with response options ranging from 1—totally unsatisfied to 5—totally satisfied.

We identified that more than half of the students surveyed were satisfied with their physical health, but that almost 30% were unsatisfied with their mental health. Furthermore, more than 50% were unsatisfied with their relationships with friends and colleagues, while 57% were satisfied with their relationships with family members. The responses are presented in Table 3.

### 3.4. Online Activity

During the present research, the academic activities of dentistry students were developed online. The majority of the students connected to the online platforms from their homes (n = 295, 97%), and a low percentage of students indicated that they participated in online activities from other places, where the internet connection was better than that at their homes (n = 8, 3%).

The need to move teaching activities to an online environment resulted in determination on the part of the universities to find platforms quickly in order to carry out courses and practical activities in the best possible conditions. Students were asked what platforms they used most often to conduct various school activities, such as those used for online connections for theoretical or practical meetings, the completion of assignments and their submission to teachers, and online courses.

A total of 63 students (20.70%) declared that they used the Microsoft Teams University Platform for their academic activity; more than half of the respondents indicated that they used Zoom, Google Meetings, and Skype (n = 202, 66.40%) more often for their online activity, and a small number of them (n = 39, 12.80%) reported that they usually preferred using other ways of communicating with colleagues and teachers (e.g., via text messages, emails, etc.). Moreover, when communicating with their colleagues regarding their academic tasks, dentistry students preferred to use social platforms (n = 299, 98.40%) or Microsoft Teams (n = 5, 1.6%).

Where communication with their teachers was concerned, students declared that they predominantly used Microsoft Teams (n = 140, 46.10%), social networks (n = 77, 25.3%), email (n = 50, 16.40%), or communicated through faculty administration staff (n = 37, 12.2%).

Most students preferred to use laptops for online activities (n = 191, 62.80%). A lower percentage said they used their smartphones to connect (n = 102, 33.60%), and an even smaller percentage said they participated in online classes using a desktop computer (n = 11, 3.60%).

A series of sixteen items were constructed in order to identify dental students’ opinions about online education. The results showed that over two-thirds of the respondents (79.90%) believed that their practical training had been affected due to the use of online activity and that not all subjects could be taught online (73.90%). We also identified that more than half of the students disagreed with the following statements: that the didactic activity was more interactive online than within the institution (44.60% totally disagreed and 18.80% disagreed with the item); that they had preference for online activity over the type carried out within the institution (51.20% totally disagreed and 14.20% disagreed with the item), and that lectures and practical stages were more interesting online.

Regarding the involvement of teachers, students appreciated that the academic staff made the courses available to students in the digital format, but they considered that the teachers were not well-trained in using the online platforms and audio–video tools.

The analysis of the responses proved that online participation did not encourage students to be active; we found that only 8.80% of students connected with a video-camera. However, 22.40% of students agreed that they could focus better on new information than when they attended the course/laboratory in person.

Two items were used to evaluate students’ opinions about the amount of time available for study and hobbies during the restrictions imposed by the COVID-19 pandemic. The results showed that more students indicated that they invested time in hobbies and recreational activities than in studying or academic tasks. The detailed results are presented in Table 4.

### 3.5. Online Evaluation

The e-learning processes and online evaluations were two of the most challenging aspects during the first months of the pandemic, because the teaching staff had to develop and use different types of instruments. Therefore, the students had to adjust to the new academic methods and tools. A number of items aimed to investigate students’ opinions regarding online evaluation as compared to the classic one, as well as the ethical aspects of online evaluation methods.

The results proved that 46.20% of students totally disagreed with the statement that online oral evaluation is the most objective method, and that 40% of the respondents totally agreed with the statement that an online multiple-choice exam is the most objective method available for the evaluation of the students.

More than 60% of the respondents agreed that the most objective evaluation method was that of the multiple-choice question exam (MCQ) administered at school, with 37.30% of the total students surveyed totally agreeing with that. It is also noteworthy that almost half of the questioned students indicated that they agreed with the opinion that exclusive online assessment of students encouraged unethical behaviors. The detailed results are presented in Table 5.

Three items investigated students’ fear regarding the risk of COVID-19 infection. This survey was conducted before their return to school. The respondents had to indicate their level of agreement with the statements using a five-item Likert-type scale rated as follows: 1—strongly disagree, 2—disagree, 3—neither agree nor disagree, 4—agree, and 5—strongly agree. The responses showed that students were more afraid of being infected by patients during their internship than of contracting the virus during classes or from classmates. The frequency of answers is presented in Table 6.

### 3.6. Comparative Results Considering Gender, Environment, Chronic Disease, and Preclinical/Clinical Years

There were significant differences between women and men in terms of e-learning teaching activity. Men (Mdn = 4.00) had more positive opinions than women (Mdn = 3.00) about the internet connection quality that influenced the quality of the teaching activity (z = −4.643, *p* < 0.001).

The results of the present research showed that, unlike women (Mdn = 2.00), men (Mdn = 3.00) considered that the teaching activity was more interesting in the online environment (z = −2.906, *p* < 0.001). At the same time, men (Mdn = 2.00) were more involved in the online activity, keeping their video camera connected during the courses (z = −2.906, *p* = 0.004), as opposed to women.

Comparative analyses showed that dental students suffering from chronic diseases (Mdn = 3.00) were less satisfied with the relationships with their family members during the restrictions related to the COVID-19 pandemic (z = −3.261, *p* = 0.001) than healthy students (Mdn = 4.00). Moreover, the results of the Mann-Whitney test (z = −2.262, *p* = 0.024) showed that students suffering from chronic diseases noticed that during the pandemic they had more sleep disorders (Mdn = 4.00), unlike the rest of the students (Mdn = 3.00). At the same time, chronically ill students considered that the amount of information taught in the online activity was higher (Mdn = 4.00), unlike healthy students (Mdn = 3.00), (z = −2.390, *p* = 0.017).

Significant differences were identified between the years of study in terms of online teaching. Students in their clinical education years preferred online activities because they were more interactive, more interesting, helped them organize their time better, and they could better focus on the new information received. Additionally, older students had a higher degree of fear regarding the possibility of COVID-19 infection during practical activities within the faculty. The detailed results for these items are presented in Table 7.

The comparative analyses also showed significant differences between students from urban areas (Mdn = 3.00) and those from rural areas (Mdn = 4.00), in the sense that students from rural areas lived in a room with more people (z = −4.728, *p* = 0.000). Significant differences were also registered regarding the occupancy of the room where the students live; thus, unlike the students who shared a room with someone (Mdn = 3.00), students who lived alone (Mdn = 2.00) considered that online courses were not so interesting (z = −3.384, *p* = 0.001).

### 3.7. Correlational Results

The correlation analyses showed that there were positive correlations between students’ age and certain behaviors. We identified that as they got older, students consumed more energy drinks (r = 0.116 *, *p* = 0.044). Age correlated positively with their degree of satisfaction regarding their academic results during the pandemic, in the sense that the advanced year of study students were in, the more satisfied they were with the results they obtained (r = 0.137 *, *p* = 0.017).

Regarding the online development of teaching activities, the results showed that the older the students, the better they could focus on the new information received (r = 0.348 **, *p* < 0.001) and the more efficiently they could organize their daily schedule (r = 0.288 *, *p* < 0.001). At the same time, the more advanced year of study students were in, the more they believed that, during the pandemic, the time they spent on studying increased (r = 0.272 *, *p* < 0.001).

Additionally, age correlated positively with students’ feelings of fear, in the sense that as they got older, they were more frightened that they might become infected with COVID-19 during practical college activities (r = 0.193 **, *p* = 0.001) or from patients with whom they came into contact during practical internships (r = 0.152 *, *p* = 0.008). We identified negative correlations between the age and physical and mental health of students, in the sense that the older the students, the more they noticed that during the pandemic they had fewer appetite disorders (r = −0.118 *, *p* = 0.039), felt less depressed (r = −0.128 *, *p* = 0.026), and felt less anxious (r = 0.145 *, *p* = 0.011).

We identified that cigarette consumption correlated positively with alcohol consumption (r = 0.328 **, *p* < 0.001) and the number of people in the household (r = 0.209 **, *p* < 0.001), in the sense that—in the case of smoking students—the more the students smoked, the more alcohol they consumed, and the more people that were in the house, the more students tended to smoke. A negative correlation was identified between cigarette consumption and the amount of time spent on recreational activities during the pandemic (r = −0.119 *, *p* = 0.039), in the sense that the more students smoked, the less time they spent on hobbies.

Our results also proved a positive correlation between alcohol consumption and energy drink consumption (r = 0.132 *, *p* = 0.021), in the sense that alcohol consumption might increase in direct proportion to energy drink consumption. Moreover, alcohol consumption correlated positively with the degree of satisfaction regarding mental health (r = 0.121 *, *p* = 0.036) and the degree of satisfaction regarding relationships with family members during the pandemic (r = 0.118 *, *p* = 0.040), meaning that students with higher alcohol consumption had better relationships with family members and were more satisfied with their mental health. A strong negative correlation was identified between alcohol and drug use (r = −0.193 **, *p* = 0.001).

Strong negative correlations were revealed between the degree of satisfaction with their physical health and several items related to the physical and mental state students felt during the pandemic, such as sleep disorders (r = −0.235 **, *p* < 0.001), eating disorders (r = −0.227 **, *p* < 0.001), depression (r = −0.227 **, *p* < 0.001), anxiety (r = −0.231 **, *p* < 0.001) and boredom (r = −0.166 **, *p* = 0.004). These results proved that the more satisfied the students were with their physical condition, the less depressed, anxious, or bored they were, and the fewer sleep disorders or appetite disorders they had. The degree of satisfaction with their mental health correlated positively with the degree of satisfaction regarding their relationships with friends (R = 0.457 **, *p* < 0.001), colleagues (r = 0.373 **, *p* < 0.001), and family members (r = 0.359 **, *p* < 0.001) during the COVID-19 pandemic, in the sense that the better the relationships with colleagues, friends, or family members, the more satisfied students were with their mental health. Furthermore, the degree of satisfaction with their mental health correlated positively with the degree of satisfaction regarding the academic results students obtained during the pandemic (r = 0.371 **, *p* < 0.001), as well as their degree of concentration on the new information received in online activities (r = 0.179 **, *p* = 0.002), in the sense that the higher students’ satisfaction with their mental health, the better they could concentrate on online activities and the more satisfied they were with their obtained academic results.

Regarding the online academic activity, the correlation analyses showed that the degree of satisfaction regarding the academic results of the students obtained during the pandemic correlated positively with students’ full attendance of their courses or laboratory instruction in the online environment (r = 0.244 **, *p* < 0.001) and the students’ preference to stay connected with video cameras during the online activity (r = 0.149 **, *p* = 0.009), in the sense that the more satisfied they were with the results they obtained, the more they tended to stay connected through video during the online activity and to participate fully in the courses. We identified a positive correlation between the degree of satisfaction regarding the academic results obtained and the students’ preference for online activity to the detriment of the traditional instruction carried out within the institution (r = 0.285 **, *p* < 0.001), in the sense that the more satisfied the students were with their results, the more they preferred to stay at home and attend online courses. At the same time, significant correlations were revealed between the degree of satisfaction with the academic results obtained by the students during the pandemic and the opinion that practical activities (r = 0.317 **, *p* < 0.001) and courses (r = 0.346 **, *p* < 0.001) were more interesting in the online format.

The results also proved a positive correlation between the fear of COVID-19 infection during practical activities carried out in the college setting and the fear of infection from patients (r = 0.881 **, *p* < 0.001) or from colleagues (r = 0.688 **, *p* < 0.001), in the sense that the more that students were scared of contracting the virus at their college, the more they feared that they could become infected from patients or colleagues with whom they came in contact during their teaching activities at the faculty. Moreover, a positive correlation was identified between the fear of infection with the new virus during practical activities at college and the preference for online classes (r = 0.508 **, *p* < 0.001), in the sense that the more scared students were of COVID-19 infection, the more they preferred to participate in online teaching activities from home.

## 4. Discussion

Medicine, regardless of specialization, can only be learned through direct contact with patients. Therefore, during the COVID-19 pandemic, a significant problem that many university centers faced was that clinical internships in hospitals and dental clinics being deeply affected, students were not allowed to enter hospitals because university management did not want to take the risk of exposing students to the disease. Therefore, in many medical specializations, including dentistry, the activity took place in a hybrid manner or completely online.

During the period of online activity, we identified that most students connected from home using their laptops, and the majority of the students utilized their audio connection; only an exceedingly small percentage connected using a video camera. This practice limited student–teacher interaction and did not stimulate teamwork or active involvement in the virtual activity. We found that 50.60% of the students attended the course/lab in its entirety when they were connected online, meaning that not using a video connection allowed students to feel free to not always be involved in online discussions. Similar conclusions were pointed out by Jiang et al. [12] in a study conducted among Chinese dental students. The authors showed that during online classes, dental students preferred lecture-based learning and case-based learning more than problem-based learning (PBL), team-based learning, or research-based learning, showing that students preferred a more traditional teaching process and passive participation during online classes. In congruency with these results, Mukhtar et al. also recommended online modalities with a clear lesson plan that reduced cognitive load and increased interaction between students or between students and teachers [34]. However, the pandemic forced the revolutionization of the dental education system through the use of technology, as teachers were determined to find ways to help students adjust to the online learning challenges.

We found that students were concerned about the impact of online classes on their level of knowledge and practical skills. More than two-thirds of the respondents considered that their practical training and dentistry skills were affected due to the online activity, and they considered that not all subjects can be taught online, especially during the practical stages of their program. Students in the last years of their study expressed more serious interest in maintaining their practical skills. We identified that students in the clinical years of their program preferred online activities and considered them to be more interactive, more interesting, and more helpful for them in terms of being able to organize their time better, and allowed them to better focus on the new information received in their instruction. We found that the older the students were, the better they could focus on the new information received, the more efficiently they could organize their daily schedule, and the more they believed that the time they spent on studying increased during the pandemic.

Even if online teaching is new in dental settings, the opinion of respondents proved that the majority agreed with the statement that the academic staff was well-trained to use the online platforms and audio–video tools. Studies investigating students’ opinions about how teachers mastered online teaching included a range of statements, from satisfaction with teachers’ skills to the opinion that staff could have more training in online learning. For example, Prieto et al. identified that dental students described teachers as showing good predisposition, flexibility, availability, and empathy through the course but also suggested better staff training in online learning [14]. A study conducted by Sarwar et al. [17] among dentistry undergraduate students from medical universities in Pakistan showed that the majority of respondents were dissatisfied with the institutional learning management system, the level of teachers’ training for online lectures, and the quality of the available learning resources. The authors identified that the worst rating was reported for items inquiring about the effectiveness of online classes; freshmen reported the poorest interaction with teachers and a strong disagreement with the effectiveness of online classes. Similar findings were described by Varvara et al. [35], who conducted a cross-sectional survey among Italian dentistry students across all years of study. The results proved that students appreciated the new methods and their teachers’ efforts during online meetings, and mentioned that the lack of practical training was a significant problem.

The results of the present study showed that students were concerned about unethical behaviors during online classes and unethical practices during online evaluation. More than 45% of the respondents agreed that the exclusive online assessment of students encouraged unethical behaviors. Similar findings were pointed out by Mukhtar et al. [34]. The authors conducted a survey among undergraduate dentistry students and identified that apart from the advantages, such as remote learning, comfort, and accessibility, teachers who used online learning were aware of the ineffectiveness and the level of difficulty involved in maintaining academic integrity. Ethical concerns are a major problem in medical sciences, with this problem also being identified in studies conducted within other medical specialties. Exam dishonesty appears as one of the major challenges faced with remote e-exams, as it was mentioned by more than one-third of questioned students in a survey conducted by Elsalem et al. [36].

Social distance and working from home resulted in a lot of changes regarding lifestyles and social relationships. We identified that, during the first seven months of the COVID-19 pandemic, students were more satisfied regarding their relationships with family members but less satisfied with their relationships with colleagues or friends, and that they had a higher level of concern regarding their mental health than they did for their physical health. Our results showed that students suffering from chronic diseases noticed that, during the pandemic, they had more sleep disorders and believed that the amount of information taught in the online activity was higher when compared with clinically healthy students.

The results of the present study showed that our respondents were more satisfied with their physical status than their mental status. After the first seven months of the COVID-19 pandemic, students self-rated as being more anxious, being more depressed, and having more sleep-related problems. Moreover, a large majority of students (over 80%) declared that they were concerned about their practical skills.

The correlation analyses proved that the more satisfied the students were with their physical condition, the less depressed, anxious, or bored they were, and the fewer sleep disorders or appetite disorders they had. The degree of satisfaction with their mental health correlated positively with the degree of satisfaction regarding their relationships with friends, colleagues, and family members during the COVID-19 pandemic. We also found that living in an apartment was correlated with a higher consumption of alcohol and cigarettes and less time spent on hobbies and relaxing activities. Similar findings were identified by Prieto et al. [14]. The authors showed that Chilean dental students reported more depression and anxiety. Machado et al. [15] showed that Brazilian students were found to have positive impressions despite technical problems and related stresses. Students mentioned, to a great extent, that the time available for recreational activities/hobbies had increased, and their time devoted to studying had decreased. Similar findings were presented by Prieto et al. [14] who showed that Brazilian dental students had the opportunity to spend more time with families, but that staying at home for an extended period of time made them more stressed and anxious. Among other negative aspects, the authors identified tiredness, loneliness, and the need for an appropriate workspace. Similar results were presented by Škrlec et al. [37], who identified that more than half of the Croatian dental students reported increased stress levels, decreased physical activity, increased depression, and increased anxiety, and that one-third of students were insufficiently active during the second COVID-19 lockdown.

We found that the higher satisfaction with their mental health was, the better students could concentrate on online activities and the more satisfied they were with the academic results they obtained. We can conclude that during the COVID-19 pandemic, the strong relationship between mental health, academic results, and the level of satisfaction with academic activity was very well highlighted. Thus, during the lockdown, students who found satisfaction in studying considered themselves physically and mentally healthier and found strategies to better cope with stress.

### Reflections and Planning

The present research presents a broad description of the student reality in a pandemic context, which could be relevant for detecting problems and making methodological and curricular decisions within the context of the training of future dentists. The present results are useful for students to understand the particular context in which they had to struggle, and also for university teachers in order to adapt their curricula and methods to meet students’ expectations as well as their own standards of teaching.

## 5. Strengths and Limitations of the Study

There are some strengths of the present research. The number of participants was beneficial for cross-sectional study, and important conclusions by gender, environment, year of study, and level of satisfaction with physical and mental health were able to be generated. Additionally, the results are important for both students and teachers working in dental institutions for across various issues related to online activity: academic tasks, evaluation methods, learning, relationships, mental and physical status, ethical concerns, and social aspects. Therefore, from this point of view, the present study presents important results for all actors in the academic field (i.e., students, teachers, and administrators).

The limitation of this research is due to the regional context, meaning that the results could be influenced by the number of infections in the area and the severity of the lockdown restrictions. So, when results are considered in comparative analyses, researchers must also take into consideration the type of activity (i.e., partially online or totally online) when they proceed in drawing conclusions.

## 6. Conclusions

The COVID-19 pandemic can be seen as a kickoff point for the motivation to invest in the technological innovation necessary to deliver the best possible education to our future dentists—even during the post-pandemic period—to ensure a higher level of satisfaction and stronger confidence in the preparedness of dental students for their future profession. The psychological impact of the online activity must not be neglected; teachers and psychologists must be aware of the strategies that have to be applied during online relationships in order to stimulate communication and increase the active participation of students.

## Figures and Tables

**Table 1:** Sociodemographic and medical characteristics ^1^.

Sociodemographic and Medical Characteristics	N (%)/M ± SD
Age	22.74 ± 2.62
Gender	
Male	78 (25.74)
Female	225 (74.26)
Year of study	
1	36 (11.88)
2	43 (14.19)
3	58 (19.14)
4	64 (21.12)
5	33 (10.89)
6	69 (22.77)
Living environment	
Rural	92 (30.36)
Urban	211 (69.64)
Having a chronic disease	
Yes	21 (6.93)
No	282 (93.07)
Smoking cigarettes	
Yes	94 (31.02)
No	209 (68.98)
Drinking alcohol	
Yes	85 (28.05)
No	218 (71.95)
Consuming energy drinks	
Yes	42 (13.86)
No	261 (86.14)
Consuming drugs	
Yes	11 (4.63)
No	292 (96.37)
Sharing a room during the academic year	
Yes	80 (26.40)
No	223 (73.60)
Being suspected of COVID-19 infection	
Yes	59 (19.47)
No	244 (80.53)
Being confirmed with COVID-19 infection	
Yes	12 (3.96)
No	291 (96.04)

^1^ Number of answers (N) and corresponding percentages (%)/means and standard deviations (M ± SD).

**Table 2:** Self-rated items regarding psychological status ^1^.

I Noticed that during the Pandemic/Since the Beginning of the Pandemic	1	2	3	4	5	M ± SD
I had had sleep disorders	82 (27.10)	52 (17.20)	59 (19.50)	55 (18.20)	55 (18.20)	2.83 ± 1.46
I had had appetite disorders	101 (33.30)	68 (22.40)	49 (16.20)	43 (14.20)	42 (13.90)	2.52 ± 1.42
I had felt more depressed	55 (18.20)	56 (18.50)	69 (22.80)	60 (19.80)	63 (20.80)	3.06 ± 1.39
I had felt more anxious	52 (17.20)	49 (16.20)	74 (24.40)	64 (21.10)	64 (21.10)	3.12 ± 1.37
I had felt more bored	33 (10.90)	18 (5.90)	64 (21.10)	64 (21.10)	122 (40.30)	3.74 ± 1.33

^1^ Number of answers (N) and corresponding percentages (%)/means and standard deviations (M ± SD); 1—strongly disagree, 2—disagree, 3—neither agree nor disagree, 4—agree, and 5—strongly agree.

**Table 3:** Satisfaction regarding relationships with family, friends, and colleagues and academic results ^1^.

During the Restrictions Related to the COVID-19 Pandemic, How Satisfied Are You with Your…	1	2	3	4	5	M ± SD
Physical health	17 (5.60)	39 (12.90)	67 (22.10)	101 (33.30)	79 (26.10)	3.61 ± 1.16
Mental health	39 (12.90)	54 (17.80)	113 (37.30)	60 (19.80)	37 (12.20)	3.01 ± 1.17
Relationships with friends	67 (22.10)	70 (23.10)	90 (29.70)	57 (18.80)	19 (6.30)	2.64 ± 1.19
Relationships with family members	21 (6.90)	34 (11.20)	75 (24.80)	81 (26.70)	92 (30.40)	3.62 ± 1.21
Relationships with colleagues	66 (21.80)	73 (24.10)	90 (29.70)	50 (16.50)	24 (7.90)	2.65 ± 1.21
Academic results	53 (17.50)	53 (17.50)	99 (32.70)	64 (21.10)	34 (11.20)	2.91 ± 1.23

^1^ Number of answers (N) and corresponding percentages (%)/means and standard deviations (M ± SD); 1—strongly disagree, 2—disagree, 3—neither agree nor disagree, 4—agree, and 5—strongly agree.

**Table 4:** Dentistry students’ opinions regarding online activity ^1^.

Items	1	2	3	4	5	M ± SD
The didactic activity was more interactive online than within the institution	135 (44.60)	57 (18.80)	60 (19.80)	23 (7.60)	28 (9.20)	2.18 ± 1.32
The academic staff made the courses in digital format available to students	2 (0.70)	22 (7.30)	42 (13.90)	96 (31.70)	141 (46.50)	4.16 ± 0.96
The online activity took place without interrupting the internet connection	63 (20.80)	52 (17.20)	89 (29.40)	53 (17.50)	46 (15.20)	2.89 ± 1.33
I preferred the online activity to the one carried out within the institution	155 (51.20)	43 (14.20)	38 (12.50)	23 (7.60)	44 (14.50)	2.20 ± 1.48
The duration of the online courses/laboratories was in accordance with the schedule	19 (6.30)	35 (11.60)	61 (20.10)	99 (32.70)	89 (29.40)	3.67 ± 1.19
During the online activity I preferred to stay connected through video	144 (47.50)	76 (25.10)	57 (18.80)	14 (4.60)	12 (4.00)	1.92 ± 1.09
Carrying out online activities determined me to organize my daily schedule in a better way	64 (21.10)	42 (13.90)	58 (19.10)	34 (11.20)	105 (34.70)	3.24 ± 1.55
In online activities I could focus better on new information than when I attended the course/laboratory	133 (43.90)	43 (14.20)	59 (19.50)	30 (9.90)	38 (12.50)	2.33 ± 1.43
I believe that online teaching was as effective as teaching within the institution	136 (44.90)	60 (19.80)	42 (13.90)	29 (9.60)	36 (11.90)	2.23 ± 1.41
The academic staff were well trained to use the online platforms and audio-video tools	57 (18.80)	76 (25.10)	77 (25.40)	50 (16.50)	43 (14.20)	2.82 ± 1.30
I believe that all subjects could be taught online	186 (61.40)	38 (12.50)	30 (9.90)	17 (5.60)	32 (10.60)	1.91 ± 1.37
The amount of information taught in the online activity was higher	36 (11.90)	39 (12.90)	130 (42.90)	52 (17.20)	46 (15.20)	3.10 ± 1.17
I consider that my practical training had been affected due to the online activity	16 (5.30)	10 (3.30)	26 (8.60)	19 (6.30)	232 (76.60)	4.46 ± 1.12
I always attended the course/lab in its entirety when I connected online	20 (6.60)	41 (13.50)	72 (23.80)	75 (24.80)	95 (31.40)	3.60 ± 1.23
The courses were more interesting online	101 (33.30)	54 (17.80)	75 (24.80)	29 (9.60)	44 (14.50)	2.54 ± 1.40
Practical activities/labs were more interesting online	177 (58.40)	50 (16.50)	41 (13.50)	15 (5.0)	20 (6.60)	1.84 ± 1.22
I believe that during the pandemic, the time given to study increased	74 (24.40)	57 (18.80)	77 (25.40)	44 (14.50)	51 (16.80)	2.80 ± 1.39
I believe that during the pandemic, the time given to recreational activities/hobbies increased	39 (12.90)	46 (15.20)	70 (23.10)	72 (23.80)	76 (25.10)	3.33 ± 1.34

^1^ Number of answers (N) and corresponding percentages (%)/means and standard deviations (M ± SD).; 1—strongly disagree, 2—disagree, 3—neither agree nor disagree, 4—agree, and 5—strongly agree.

**Table 5:** Self-rated items regarding students ‘opinions related to online evaluation ^1^.

Items	1	2	3	4	5	M ± SD
Online student assessment was more objective	77 (25.40)	35 (11.60)	93 (30.70)	30 (9.90)	68 (22.40)	2.10 ± 1.27
I consider that the most objective evaluation method was the online oral exam	140 (46.20)	61 (20.10)	54 (17.80)	27 (8.90)	21 (6.90)	2.10 ± 1.27
I consider that the most objective evaluation method was the online MCQ	32 (10.60)	28 (9.20)	57 (18.80)	63 (20.80)	123 (40.60)	3.71 ± 1.35
I consider that the most objective evaluation method was the summative one (several assignments during the semester, both online and in class)	55 (18.20)	39 (12.90)	106 (35.00)	64 (21.10)	39 (12.90)	2.97 ± 1.25
I consider that the most objective evaluation method was the oral one, at school	76 (25.10)	58 (19.10)	70 (23.10)	46 (15.20)	53 (17.50)	2.80 ± 1.41
I consider that the most objective evaluation method was the one through the MCQ, at school	24 (7.90)	25 (8.30)	67 (22.10)	74 (24.40)	113 (37.30)	3.74 ± 1.25
I believe that exclusive online assessment of students encouraged unethical behaviors	49 (16.20)	32 (10.60)	83 (27.40)	60 (19.80)	79 (26.10)	3.29 ± 1.38

^1^ Number of answers (N) and corresponding percentages (%)/means and standard deviations (M ± SD); 1—strongly disagree, 2—disagree, 3—neither agree nor disagree, 4—agree, and 5—strongly agree.

**Table 6:** Items investigating the fear of COVID-19 infection ^1^.

Items	1	2	3	4	5	M ± SD
I was afraid that I might become infected with COVID-19 during practical activities at college	86 (28.40)	54 (17.80)	57 (18.80)	39 (12.90)	67 (22.10)	2.82 ± 1.51
I was afraid that I might become infected with COVID-19 from patients with whom I came in contact during internships	75 (24.80)	53 (17.50)	51 (16.80)	46 (15.20)	78 (25.70)	2.99 ± 1.53
I was afraid that I might become infected with COVID-19 from the colleagues with whom I came in contact at college	110 (36.30)	50 (16.50)	61 (20.10)	33 (10.90)	49 (16.20)	2.54 ± 1.47

^1^ Number of answers (N) and corresponding percentages (%)/means and standard deviations (M ± SD); 1—strongly disagree, 2—disagree, 3—neither agree nor disagree, 4—agree, and 5—strongly agree.

**Table 7:** Opinions of dental students regarding online activity. Differences by preclinical versus clinical years ^1^.

Items	Mann-Whitney	Results		Median	
	Mann-Whitney U	z	*p*	Preclinical	Clinical
The didactic activity was more interactive online than within the institution	8292.00	−4.286	0.000	1.00	2.00
I preferred the online activity to the one carried out within the institution	8791.00	−3.670	0.000	1.00	2.00
Carrying out online activities determined me to better organize my daily schedule	7667.00	−5.039	0.000	3.00	4.00
In online activities I could focus better on new information than when I attended the classroom/laboratory	8639.00	−3.789	0.000	1.00	3.00
I always followed the course/lab in its entirety when I connected online	9186.00	−2.973	0.003	3.00	4.00
The courses were more interesting online	8463.50	−3.955	0.000	2.00	3.00
I was afraid that I might become infected with COVID-19 during practical activities at the faculty	9691.00	−2.269	0.023	2.00	3.00

^1^ Mann-Whitney analysis results.

## Data Availability

The data presented in this study are available on request from the corresponding author.
